# SP-D counteracts GM-CSF-mediated increase of granuloma formation by alveolar macrophages in lysinuric protein intolerance

**DOI:** 10.1186/1750-1172-4-29

**Published:** 2009-12-23

**Authors:** David N Douda, Nicole Farmakovski, Sharon Dell, Hartmut Grasemann, Nades Palaniyar

**Affiliations:** 1 Lung Innate Immunity Research, Program in Physiology and Experimental Medicine, Research Institute, The Hospital For Sick Children, Toronto, Ontario, M5G 1X8, Canada; 2 Division of Respiratory Medicine, The Hospital For Sick Children, Toronto, Ontario, M5G 1X8, Canada; 3 Department of Laboratory Medicine and Pathobiology, Faculty of Medicine, University of Toronto, Toronto, Ontario, M5G 1X8, Canada

## Abstract

**Background:**

Pulmonary alveolar proteinosis (PAP) is a syndrome with multiple etiologies and is often deadly in lysinuric protein intolerance (LPI). At present, PAP is treated by whole lung lavage or with granulocyte/monocyte colony stimulating factor (GM-CSF); however, the effectiveness of GM-CSF in treating LPI associated PAP is uncertain. We hypothesized that GM-CSF and surfactant protein D (SP-D) would enhance the clearance of proteins and dying cells that are typically present in the airways of PAP lungs.

**Methods:**

Cells and cell-free supernatant of therapeutic bronchoalveolar lavage fluid (BALF) of a two-year-old patient with LPI were isolated on multiple occasions. Diagnostic BALF samples from an age-matched patient with bronchitis or adult PAP patients were used as controls. SP-D and total protein content of the supernatants were determined by BCA assays and Western blots, respectively. Cholesterol content was determined by a calorimetic assay or Oil Red O staining of cytospin preparations. The cells and surfactant lipids were also analyzed by transmission electron microscopy. Uptake of Alexa-647 conjugated BSA and DiI-labelled apoptotic Jurkat T-cells by BAL cells were studied separately in the presence or absence of SP-D (1 μg/ml) and/or GM-CSF (10 ng/ml), *ex vivo*. Specimens were analyzed by light and fluorescence microscopy.

**Results:**

Here we show that large amounts of cholesterol, and large numbers of cholesterol crystals, dying cells, and lipid-laden foamy alveolar macrophages were present in the airways of the LPI patient. Although SP-D is present, its bioavailability is low in the airways. SP-D was partially degraded and entrapped in the unusual surfactant lipid tubules with circular lattice, *in vivo*. We also show that supplementing SP-D and GM-CSF increases the uptake of protein and dying cells by healthy LPI alveolar macrophages, *ex vivo*. Serendipitously, we found that these cells spontaneously generated granulomas, *ex vivo*, and GM-CSF treatment drastically increased the number of granulomas whereas SP-D treatment counteracted the adverse effect of GM-CSF.

**Conclusions:**

We propose that increased GM-CSF and decreased bioavailability of SP-D may promote granuloma formation in LPI, and GM-CSF may not be suitable for treating PAP in LPI. To improve the lung condition of LPI patients with PAP, it would be useful to explore alternative therapies for increasing dead cell clearance while decreasing cholesterol content in the airways.

## Background

Pulmonary alveolar proteinosis (PAP) syndrome is thought to occur due to multiple causes including genetic defects [1-3], immune deficiencies [4], malignancies [5,6] and infection [7]. Patients with PAP have milky alveolar infiltrates that often contain excess surfactant material and large numbers of white blood cells. PAP is usually treated by whole lung lavage (WLL) [8-12] or with GM-CSF administration [13-16]. GM-CSF is a hematopoietic growth factor known to stimulate stem cells to proliferate into granulocytes or monocytes [17], promote differentiation of monocytes into alveolar macrophages [18-20], and increase the catabolism within alveolar macrophages [21,22], and increase the innate immune potential of neutrophils [23]. Therefore, GM-CSF is an important cytokine that could regulate PAP at multiple levels.

PAP-like condition is also reported in surfactant protein D (SP-D) deficient mice [24-26]. SP-D is an innate immune collectin that is present in lungs and other mucosal surfaces [27]. It opsonizes pathogens and dying cells, and enhances their uptake by the alveolar macrophages [28,29]. SP-D deficient mice also accumulate lipid-laden foamy macrophages [25,26] and apoptotic cells in their lungs [30]. Moreover, transgenic over-expression of SP-D [31] or administration of recombinant fragments of SP-D [30] has been shown to enhance immune function in the lungs in mice. Therefore, SP-D and GM-CSF could significantly enhance the innate immune functions of alveolar macrophages.

PAP is one of the deadliest phenotypes seen in Lysinuric protein intolerance (LPI) [32-34]. LPI is an autosomal recessive disorder characterized by mutations in the SLC7A7 (solute carrier family 7, member 7) gene, which encodes a dibasic cationic amino acid transporter, y+LAT1 [35,36]. Mutant SLC7A7 proteins cause defective transport of the cationic amino acids arginine, lysine, and ornithine [35-38]. PAP develops in some LPI patients and appears to be different from idiopathic PAP [32]. PAP in LPI presents with large numbers of cholesterol crystals and granulomas [32,33,39]. The precise cause or appropriate treatment options for the clinical presentation of PAP in LPI is currently unknown. Since LPI is not a well-characterized disease, and has multiple symptoms [37], it may have also been misdiagnosed as other conditions, and hence, the prevalence of this disease may be higher than the reported numbers [37]. Therefore, it is important to study the disease phenotypes and treatment options. We hypothesized that defective clearance of materials present in the airways contributes to the presentation of PAP in LPI patients, and treating the LPI cells with SP-D and GM-CSF could enhance the innate immune potential of these cells.

Consistent with our hypothesis, we found that SP-D and GM-CSF increase the uptake of proteins and dying cells by LPI AMs. Unexpectedly, we found that the LPI AMs spontaneously formed granulomas, *ex vivo*. Moreover, while control cells remained unchanged, the addition of GM-CSF to the LPI AM cultures resulted in a marked increase in the number of granulomatous structures. Notably, SP-D counteracted the negative effect of GM-CSF. In conclusion, although GM-CSF may have therapeutic advantage in certain types of PAPs, it may not be suitable for treating PAP of the LPI patients.

## Materials and methods

### Mutation analysis

SLC7A7 gene mutation analysis was performed by Dr. Ginafranco Sebastio in Naples, Italy. ABCA3, SP-B and SP-C genes were sequenced and analyzed by Ambrey Genetics.

### Reagents

All buffer salts and reagents were obtained from Sigma unless otherwise stated. SP-D was isolated from BALF of an adult PAP patient as described previously [40,41]. Recombinant GM-CSF expressed in yeast (Leukine) was produced by Berlex (Seattle, WA). All cell culture media were obtained from Invitrogen (Carlsbad, CA).

### Collection of WLL and BALF

Therapeutic WLL was performed in the LPI patient or idiopathic PAP patient, and the BALF samples were colleted in 100 ml vials. Diagnostic BALF sample was also obtained from another airway disease patient. BALF was filtered with nylon mesh and centrifuged at 200 × g for 10 min at 4°C. The pellets containing the cells were then washed twice with Hank's Buffered Salt Solution (HBSS). The supernatant was centrifuged again at 10,000 × g for 40 min at 4°C to collect surfactant lipid pellets, which were stored at -80°C until further analyses.

### Analysis of the BALF

BCA^® ^Protein Assay kit from Pierce (Rockford, IL) was used for measuring the protein concentrations in the pre-filtered BALF from each collection vial according to the manufacturer's instructions. SP-D concentration levels and degradation were assessed in the BALF from the LPI patient by Western blotting using anti-human SP-D rabbit sera as described previously [42]. An aliquot of the BALF from each collectin tube was immediately mixed with 0.2% (w/v) Trypan Blue solution and counted using a Hemocytometer at 20× or 40× normal magnification. The amount of cholesterol present in the BALF samples were assessed using the Cholesterol Assay Kit (Cayman Chemical Co., Ann Arbor, MI).

### Histological staining

Cytospin preparations were made using 50 and 100 μl BALF, cells were air dried and subjected to Hemacolor^® ^staining (EMD Chemicals, Inc, Gibbstown, NJ) for histological analysis. Other cytospin preparations were fixed with 2% (v/v) paraformaldehyde (PFA) in PBS for 1 h at 4°C, and were subjected to staining with Oil Red O (Polysciences, Inc, Washington, PA) for lipid analysis of foamy macrophage cells. Oil Red O stock solution was prepared by dissolving 0.5% (w/v) Oil Red O powder in isopropanol at 37°C. This stock solution was then diluted to 60% (v/v) with dH_2_O to make the working solution. Cytospin preparation of cells were rinsed with 60% (v/v) isopropanol, and stained with freshly prepared Oil Red O working solution for 15 min and counterstained with Hemacolor Solution 3 for nuclei. Cells were imaged using Leitz Laborlux D microscope with Leica IM 50 image manager software.

### Electron microscopy

Cell pellets and surfactant lipid pellets obtained from 200 × g and 10,000 × g centrifugation of BALF, respectively, were fixed for at least 18 h at 4°C with 2% (v/v) paraformaldehyde (PFA) and 4% (v/v) glutaraldehyde (Electron Microscopy Sciences, Fort Washington, PA) in a buffer containing 50 mM HEPES (pH 7.4), 150 mM NaCl, and 1 mM CaCl_2_. Samples were then processed, stained with 2% (w/v) uranyl acetate and analyzed by transmission electron microscopy (JEOL TEM 1011) with AMT Image Capture Engine software at 2,000-40,000 times normal magnification.

### Cell culture

Cell pellets obtained from 200 × g centrifugation of the BALF were immediately re-suspended in Dulbecco's Modified Eagle's Medium (DMEM) supplemented with 10% (v/v) heat-inactivated Fetal Bovine Serum (FBS) with 100 U/ml penicillin, 100 μg/ml streptomycin, and 2.5 μg/ml Fungezone. Cells were then allowed to adhere to chamber slides or 24- or 48-well culture plates for 1 h, and non-adherent cells were washed. Cells were cultured in the same media, and subsequently, the media was changed to Macrophage Serum-Free Media (MSFM), a specific media available for culturing macrophage cells (Invitrogen), and does not require supplementation with serum, prior to the experiments. The Jurkat T cell line was maintained in RPMI containing 10% (v/v) FBS.

### Induction of apoptosis

Jurkat T cells were first labelled with 2 μl/ml DiI membrane dye (Invitrogen) in MSFM for 4 min at 37°C, followed by three washes with HBSS. Apoptosis was induced by UVC irradiation for 30 s using UV Stratalinker 2400 (120 J/cm^2^). Cells were then placed in the humidified incubator at 37°C for 2 h in the absence or presence of SP-D (5 μg/ml).

### BSA/Apopotic cell uptake assay

Cells were incubated first in MSFM overnight prior to the experiment. Cells were then treated with SP-D (1 μg/ml) for 4 h, and GM-CSF (10 ng/ml) for 30 min prior to the experiment. Alexa-647 conjugated BSA was added to the culture for 30 min, at which point the cells were fixed with 2% (v/v) PFA for 10 min at room temperature, followed by fresh 2% (v/v) PFA for 1 h at 4°C. DiI-labelled apoptotic Jurkat T cell uptake assay was performed in the same manner as BSA using the Jurkat cells 2 h after the induction of apoptosis (1:5 of apoptotic cells:macrophages).

### Fluorescence microscopy and image analysis

Images for the uptake assays, and granuloma assay were captured using Zeiss Axiovert 200 M fluorescence microscope with Volocity software. Measurement of cell area, color (label-specific gray levels) and size was performed using Volocity software.

### Granuloma formation assay

Cells were incubated in the presence or absence of SP-D (1 μg/ml) and/or GM-CSF (10 ng/ml) for 29 days *ex vivo*. Culture media was replaced with a fresh media with the addition of appropriate reagents (SP-D and/or GM-CSF) every 3-5 days. After 28 days, the granulomas started to dislodge from the substratum.

### Statistical analysis

A two-tailed student's t-test was performed using Microsoft Excel on Oil Red O positive cell counting and apoptotic Jurkat cell uptake analysis. Protein uptake assay, and cell shape and size were analyzed using Dunnett's multiple mean comparison tests using JMP statistical analysis software (Version 5.0; SAS Institute Inc.). A p-value was set at 0.05 for statistical significance. Non-linear regression analysis and comparisons of regression lines by ANOVA and F-test was performed using GraphPad PRISM statistical analysis software (Version 4.0; GraphPad Software).

## Results

### LPI and control patients

A Canadian boy with Dutch ancestry was diagnosed with LPI at 9 months of age. His symptoms included feeding difficulty, vomiting, hepatomegaly and failure to thrive. Symptoms improved after initiating dietary therapy with protein restriction and citrulline supplementation. However, a progressive productive cough developed by age 18 months. Chest X-rays showed an interstitial pneumonitis pattern and bronchiolar lavage fluid confirmed the presence of Periodic acid-Schiff (PAS) stain positive staining alveolar macrophages consistent with the diagnosis of PAP. The patient, however, did not have mutations in ABCA3, SP-B, or SP-C. Despite appropriate dietary therapy and treatment of gastro-esophageal reflux, the boy's respiratory symptoms progressed and he developed a persistent oxygen need. A trial of systemic steroid therapy (2 mg/kg body weight) was ineffective. At 2 1/2 years of age referral was made to our pediatric respiratory medicine service. Since WLL is associated with significant morbidity and technically difficult in small children, a three-month trial of inhaled GM-CSF (250 μg once daily delivered through a pari-star nebulizer with a mouthpiece) was instituted. The boy's respiratory deterioration continued. Therefore, a WLL procedure was performed after the discontinuation of GM-CSF treatment. After the procedure, there seemed to be some initial clinical improvement, but the respiratory symptoms relapsed within 3 to 5 days. His disease progressed to the point of respiratory failure requiring intubation and mechanical ventilation despite two further lung lavages. A lung biopsy only confirmed the diagnosis of PAP and interstitial pneumonitis secondary to LPI. The presence of cholesterol granulomas in the airways was also confirmed by the biopsy. The boy was unresponsive to further courses of high dose pulse intravenous corticosteroid therapy and eventually died.

Diagnostic BALF samples from a patient who had bronchitis of unknown etiology and no specific airways disease were used as a control. In some experiments, we also used BALF samples obtained from adult idiopathic PAP patient.

### BALF contains high levels of protein and dying cells

**Figure 1:**
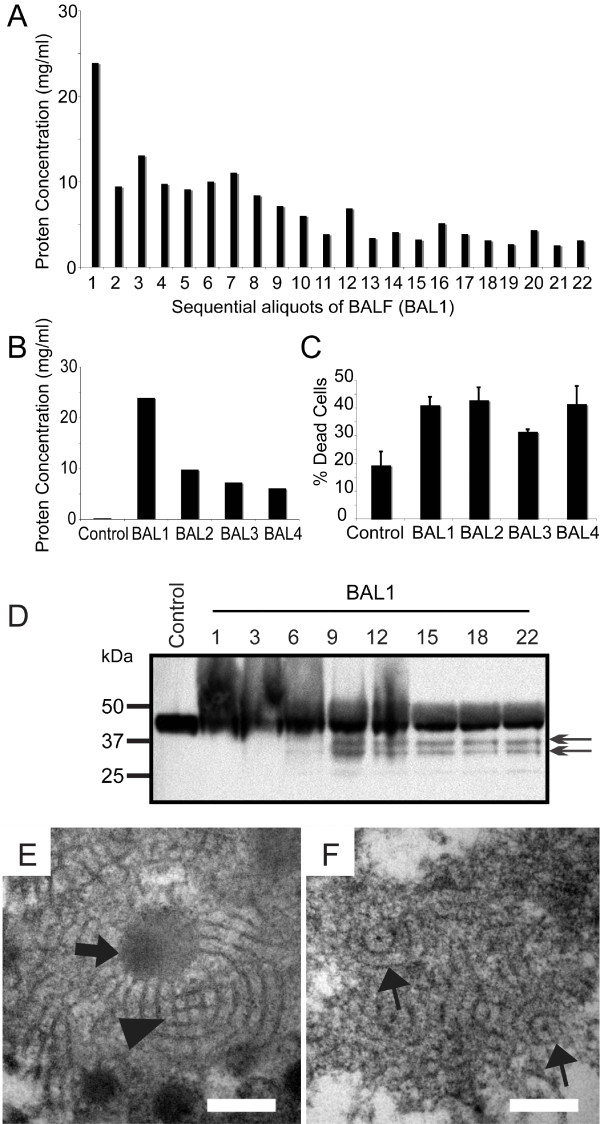
**The airways of LPI lungs with PAP contain high levels of protein, dying cells and has low bioavailability of SP-D**. (A) BCA assay results showing the protein concentrations of the BALF in each collectin vial (left lung). Protein concentration gradually decreased with subsequent lavages. No noticeable changes in protein concentrations occur after about 10 washes with 100 ml saline each. (B) Protein concentrations determined by BCA assay on the samples present in the first collection vial of four lavages conducted on separate occasions. BAL1, left lung; BAL2-4, right lung. Control sample was obtained from a diagnostic BAL from a patient with bronchitis. (C) Trypan blue exclusion assay showing the dead cells present in the BALF. Values represent the % of dead cells present in the total cells isolated from the BALF. (D) Western blot for SP-D. Initial lavage samples contained slowly migrating SP-D (smear). Later samples show multiple SP-D bands with lower molecular weights suggestive of degraded SP-D fragments. (E and F) LPI lung surfactant contains altered atypical tubular myelin with circular lattice. TEM analysis was performed on lipid pellet isolated from 10,000 × g centrifugation of cell-free LPI BALF. (E) High abundance of amorphous electron dense lipid-droplet like material (arrow) and some classical SP-A-mediated tubular myelin structures with square lattice-like structures are seen (arrowhead). (F) Large numbers of atypical tubular myelin structures with circular lattices and a central electron dense dot are also detected (~90 nm diameter; arrows). Bar, 100 nm (E and F).

To determine the alterations in the proteins present in the patient's airways, we analyzed the lavage samples by BCA assay. Large quantities of proteins were present in the patient's airways, and the protein content gradually decreased in successive collection aliquots (Figure 1A). Compared to the control BALF, the protein concentration in the airways of the LPI patient was over 100-fold higher in the first lavage (Figure 1B). Although repeated BAL procedures across different procedural days (BAL2, 3, and 4) and different lobes reduced the protein concentration, they remained over 30-fold higher compared to control (Figure 1B). In addition to increased protein concentration, large numbers of dead/dying cells were present in these samples as revealed by Trypan blue exclusion assays (Figure 1C). On average, approximately 40% dying cells were present in these LPI BALF samples.

### Reduced bioavailability of SP-D in LPI PAP surfactant

The role of SP-D in human PAP is not well understood. Using multiple LPI PAP lavage samples from the patient, we first determined whether there were any alterations in SP-D. Western blots detected SP-D that was either modified or tightly associated with other components present in the airways (upward smear) as well degraded SP-D (low molecular weight bands) (Figure 1D). This suggested that the bioavailability of functional SP-D would be low in these airways. To further assess this point, we analyzed the surfactant lipids isolated from the supernatant obtained after removing the cell pellet. The surfactant lipid isolation was performed by a subsequent centrifugation of this supernatant at high-speed (10,000 × g). We first examined the surfactant lipid structure in detail. Lattice-like tubular myelin structure was detected in the surfactant lipid (Figure 1E, arrowhead). There were also many amorphous electron-dense structures present (Figure 1E, arrow). Notably, the PAP surfactant also contained unusual forms of tubular myelin with circular lattices (Figure 1F). This circular tubular myelin had the cross-sectional diameter of approximately 90 nm, with electron dense "spot" in its centre, a structure similar to that of SP-D mediated tubular myelin [43]. These unusual structures indicated that SP-D is likely trapped inside these tubular structures. The presence of atypical circular tubular myelin and the cleaved/modified collectin suggested that the bioavailability of SP-D would be low in these airways.

### LPI PAP BALF contains abundant cholesterol and foamy cells

**Figure 2:**
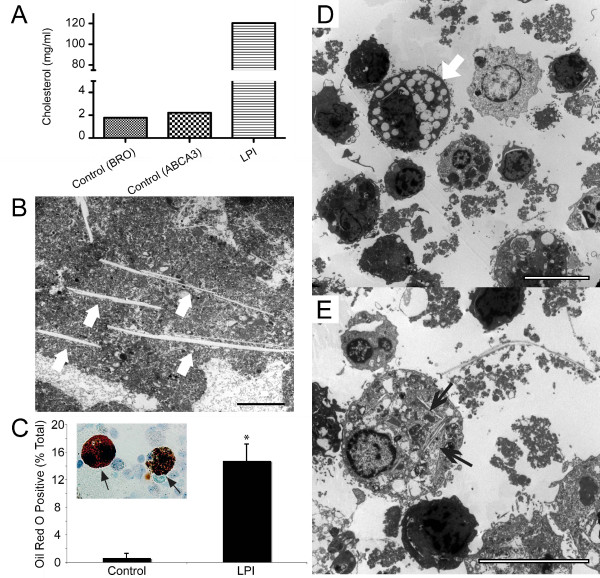
**The airways of LPI lungs contain high amount of cholesterol, cholesterol needles and foamy macrophage cells**. (A) Cholesterol assay revealed over 60-fold increase in cholesterol level of LPI BALF compared to controls. Control samples were obtained from diagnostic BALF of a patient with bronchitis (BRO) and a patient who carries mutation in ABCA3 transporter (ABCA3). (B) Several needle-like structures reminiscent of cholesterol crystals (arrows) are present within LPI surfactant lipid. Bar: 10 μm. (C) Quantitative analysis show that a significantly higher numbers of Oil Red O positive cells are present in LPI BALF compared to control BALF. The inset image shows representative large and foamy Oil Red O positive cells (arrows). Data are expressed as the mean percentage of positive cells in total cells ± SEM. Student's t-test was performed with * p < 0.05. (D) Transmission electron microscopy of cell pellets obtained from 200 × g centrifugation of LPI BALF shows the presence of lipid-laden foamy macrophage cells (arrow) and dying cells and/or lymphocytes in LPI BALF. (E) Many of the alveolar macrophages contained needle-like cholesterol crystals in their cytoplasm. Several long extracellular cholesterol needles are also visible (top right). Bar, 10 μm.

To determine the cholesterol level in the surfactant, we measured the amount of cholesterol present in the LPI BALF. The LPI BALF contained over 60-fold higher amounts of cholesterol compared to the control samples (Figure 2A). This is particularly important as serum cholesterol levels in the LPI patient were found to be in the normal range (data not shown). We also detected many needle-like cholesterol crystals in the lipid pellet, ranging from 2 μm to over 30 μm in length (Figure 2B)

To characterize the different types of immune cells present in the airways, cytospin preparations were made from BALF, fixed and stained with Oil Red O, a dye that primarily stains neutral lipids, triglycerides and lipoproteins (Figure 2C). While few cells from bronchitis control BALF were Oil Red O positive, a significantly higher percentage of cells stained positive in the LPI BALF indicating the presence of lipid-laden foamy macrophage cells (> 20-fold; p < 0.05; Figure 2C). To further confirm the nature of these cells, cells were isolated from BALF samples and examined using electron microscopy. Large numbers of vacuolated foamy macrophages, other immune cells, small-sized apoptotic cells with condensed nuclei and cellular debris were readily detected (Figure 2D). Furthermore, foamy cells that contain cholesterol crystals within their cytoplasm were often observed (Figure 2E). Large cholesterol needles were also present outside the cells in these preparations. Collectively, these results demonstrated that the LPI patient had PAP with multiple alterations potentially impairing lung function.

### LPI alveolar macrophage cells respond to SP-D and GM-CSF during uptake of protein and apoptotic cells

**Figure 3:**
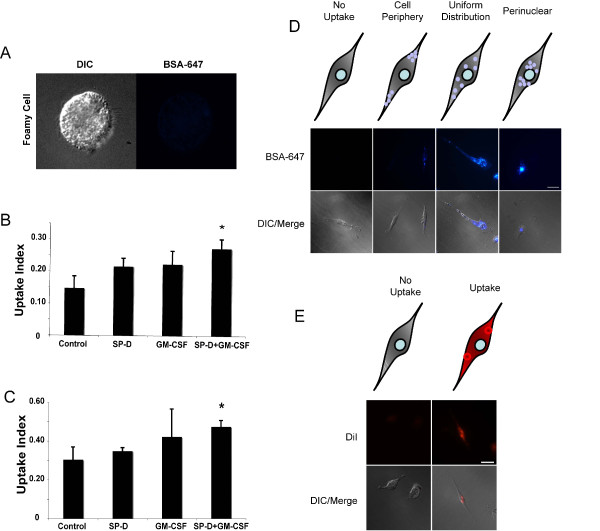
**Adherent viable cells isolated from LPI BALF respond to SP-D and GM-CSF and increase uptake of protein and dying cells**. (A) LPI BALF contained foamy macrophage cells that did not take up protein. These cells did not survive even an over night culturing, and thus, was not included in the quantification of uptake index. (B) Pre-treatment of primary cells isolated from the BALF with SP-D and GM-CSF facilitate the transport of Alexa-647 conjugated BSA to perinuclear regions. Cells were quantified and presented as means ± SEM of percentage of cells. n = three independent experiments. At least 70 cells were counted for each condition. Uptake index represents the proportion of cells that have Alexa-647 signal in the perinuclear region out of total cell present. The means were compared with respective controls using Dunnett's multiple mean comparison procedure (* p < 0.05). (C) Presence of SP-D and GM-CSF increase the uptake of apoptotic cells by LPI BAL cells. n = three independent experiments. The uptake index represents the proportion of cells that show uptake of apoptotic material out of total cell present. The means were compared with respective controls using Dunnett's multiple mean comparison procedure (* p < 0.05). (D) Representative images showing different categories of cells found 30-min after BSA feeding. (E) Representative images showing the two categories: No uptake; Uptake, where internalized apoptotic bodies/cells were present inside the macrophages.

We then sought to determine whether SP-D and/or GM-CSF would enhance the uptake of various components typically present in the PAP airways by LPI alveolar macrophages. To assess this, we tested whether the uptake of protein (Alexa-647 conjugated BSA) and apoptotic Jurkat cells could be increased in the presence of SP-D and/or GM-CSF (Figure 3). Among the cultured BAL cells, "healthy" cells with alveolar macrophage morphology readily adhered to the culture plates for long period of time. However, the foamy cells did not survive the culturing conditions, and did not show detectable uptake of labeled protein (Figure 3A); thus, these healthy cells were used for the subsequent experiments (Figure 3B-E). For this, BAL cells that were collected by centrifugation of BALF at low speed (200 × g) and plated in chamber slides were cultured. In the uptake assays, these cells were pre-incubated with SP-D (1 μg/ml) for 4 h, and/or with GM-CSF (10 ng/ml) for 30 min prior to feeding (Figure 3B and 3D). In general, qualitative examination showed that pre-incubation of cells with SP-D and GM-CSF enhanced the uptake of Alexa-647 BSA by the BAL cells. In order to quantify the uptake of Alexa-647 BSA, we manually counted different types of BSA uptake of randomly selected cells from the images (double blinded) using a sorting system. The counting categories consisted of "Cell Periphery", "Uniform Distribution", "Perinuclear" and "No Uptake". Of those categories, "Uniform Distribution" and "Perinuclear" categories were considered separate because the internalized materials are processed within the cells by fusing with lysosomes, which are primarily located in large numbers in the perinuclear regions of the cells [44]. We inferred that the increase in the number of cells in "Perinuclear" category would be an increase in the processing rate of the internalized material. Compared with buffer control, co-incubation of cells with both SP-D and GM-CSF significantly increased the proportion of cells belonging to perinuclear category (p < 0.05; Figure 3B). Therefore, we infer that the presence of both SP-D and GM-CSF enhances the uptake/clearance of proteins by LPI macrophages.

SP-D deficiency results in the accumulation of apoptotic cells, *in vivo *[30], and PAP airways contain increased number of dead cells (Figures. 1 and 2). Thus, we next asked whether SP-D and/or GM-CSF could enhance the clearance of apoptotic cells by the cells isolated from the LPI BALF (Figure 3C and 3E). These cells were incubated with apoptotic Jurkat T cells for 4 h in the presence/absence of SP-D and/or GM-CSF. SP-D was pre-incubated with apoptotic cells after the induction of apoptosis with UV irradiation. We manually quantified the images of cells as either "Uptake" where the cells have taken up apoptotic cell material, and "No Uptake". Manual counting of randomly selected images (double blinded) revealed that, like BSA uptake, the uptake of apoptotic cells/bodies was significantly increased when macrophages were incubated with SP-D coated apoptotic cells in the presence of GM-CSF (p < 0.05; Figure 3C).

### SP-D and GM-CSF affect the cell size and shape

**Figure 4:**
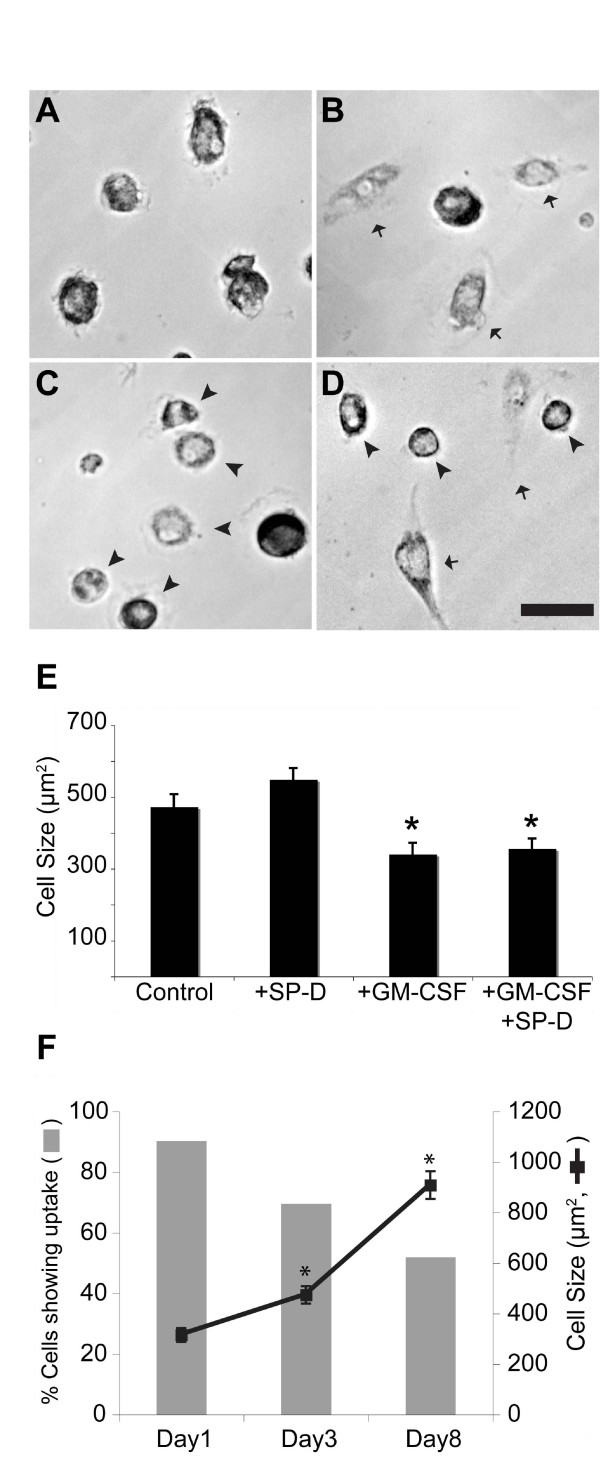
**SP-D and GM-CSF affect the shape and size of the cells**. (A-D) Adherent viable cells were grown in the absence or presence of SP-D (1 μg/ml) and/or GM-CSF (10 ng/ml) for 24 h. (A) Cells without any added proteins have relatively the same size and shape. (B) In the presence of SP-D, more cells became elongated in shape (arrows). (C) In the presence of GM-CSF, more cells became small, round or oval (arrowheads). (D) In the presence of both SP-D and GM-CSF, both populations (arrowheads, oval; arrow, elongated) are present. (E) Average cell size shown in the above conditions. Presence of GM-CSF significantly reduced cell size, reflecting the formation of small and spherical/oval cells. The sizes of at least 50 cells were measured in each category. Data are presented as means ± SEM; n = two independent experiments. The means were compared with respective controls using Dunnett's multiple mean comparison procedure (* p < 0.05). (F) Over one week time, many BAL cells changed into elongated and stopped internalizing foreign materials (labeled BSA). Number of cells that actively internalize the foreign material was quantified from the randomly selected images obtained in uptake assays. The increase in cell size was associated with decrease in % cells that internalize BSA. For percent cells that actively internalize, data are presented as the proportion of cells that were able to take up Alexa fluor 647-conjugated BSA in total number of cells present. The mean values were compared with Day1 value using Dunnett's multiple mean comparison procedure (* p < 0.05).

We next asked whether SP-D and GM-CSF alter the cells isolated from BALF. Analysis of randomly selected images obtained from the feeding experiments showed that SP-D and GM-CSF affect the shape and size (as defined by the area occupied by each cell) of the cells (Figure 4). While control cells consisted of relatively uniform shapes and sizes (Figure 4A), the presence of SP-D promoted cell spreading (Figure 4B) and GM-CSF made cells more spherical/oval in shape (Figure 4C). Presence of both SP-D and GM-CSF generated mixed populations of both elongated and spherical cells (Figure 4D). Measurement of cell size also showed that GM-CSF, both in the presence and absence of SP-D, caused a significant decrease in cell size (p < 0.05; Figure 4E). Thus, these results show that SP-D may increase cell spreading, whereas GM-CSF maintains cells in a spherical shape.

During the course of our experiments, we serendipitously discovered that the BAL cells began to progressively extend long processes, especially those cultured *ex vivo *for longer than 7 days. In agreement with this phenomenon, the cell size increased with increase in number of days (Figure 4F). Because of the change in cell shape and size, we asked whether there was an effect on the ability of these cells to internalize BSA. Quantifying BSA uptake revealed that the proportion of cells participating in the uptake of BSA decreased over time, and was inversely related to cell size (Figure 4F).

### GM-CSF increases granulomatous structure formation

**Figure 5:**
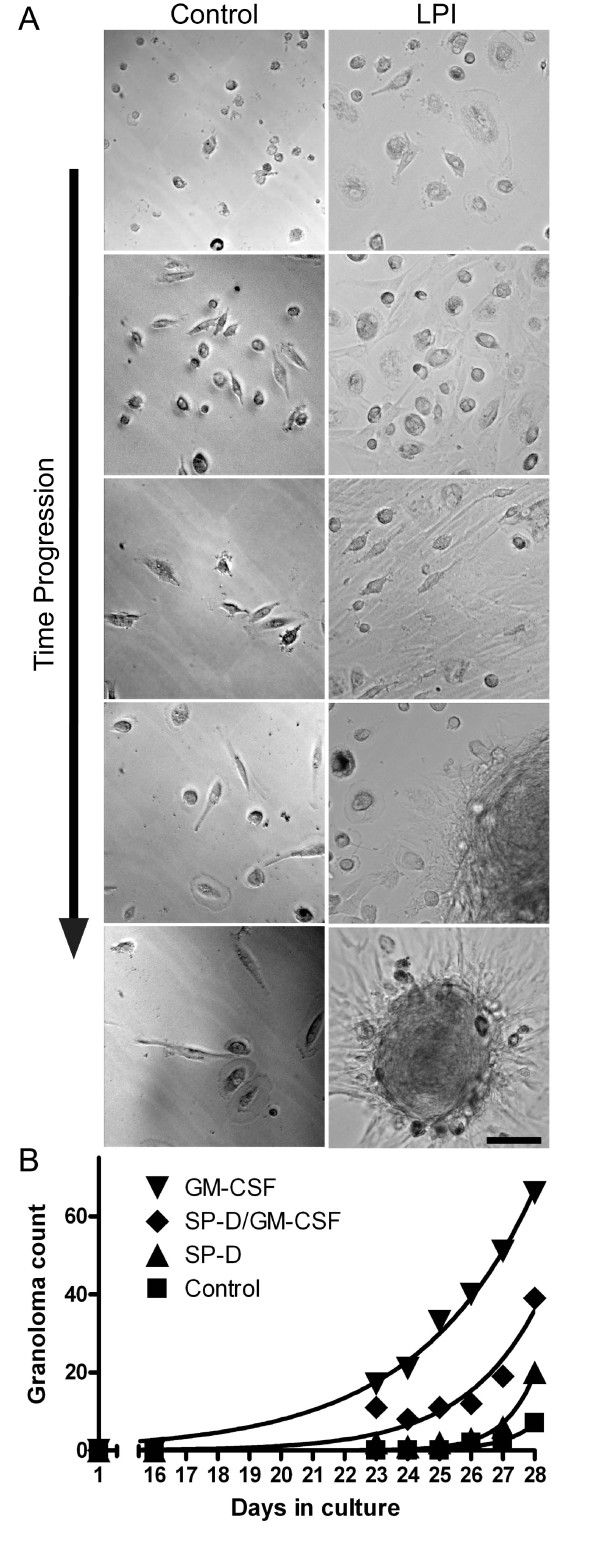
**LPI cells, but not the control cells, isolated from BALF spontaneously form granuloma**. (A) While cells from the control BALF did not elongate to a greater degree, many LPI cells became highly elongated. LPI cells then formed granulomas while the control cells remained relatively unchanged (26 days *ex vivo*). All of these cells moved to one location and eventually formed granulomas over time. The formation of these granulomas was reproducible under different culturing conditions. Images represent randomly selected cells captured every 3-4 days. Bar, 100 μm. (B) Effect of SP-D and GM-CSF on granuloma formation. Treatment of cells with GM-CSF greatly enhanced the formation of granulomatous structures whereas SP-D reduced the effect of GM-CSF-mediated granuloma formation. Non-linear regression analysis and comparison by F-test revealed statistical differences in granuloma formation over the entire experimental period among these four different treatments (p < 0.05).

Remarkably, when these BAL cells were cultured *ex vivo *for an even longer period of time (> 20 days), these cells began to form previously uncharacterized granulomatous structures (Figure 5). The formation of these structures was reproducible under different conditions. The granulomas formed *ex vivo *consisted of smaller spherical cells and elongated cells. These clearly defined cells were primarily found at the periphery of the granulomas (Figure 5A). Although the precise nature of these structures is unknown, given our observation that GM-CSF makes cells more spherical, we asked whether incubation of these cells with GM-CSF would reverse, or slow down the formation of elongated cells and attenuate or prevent granuloma formation. Surprisingly, presence of GM-CSF (10 ng/ml) dramatically increased the number of granulomatous structures present in the culture (Figure 5B). On the other hand, the control BAL cells only became slightly elongated. We performed a regression analysis on the quantification of granulomas formed in each culture and compared each condition using an F test (Figure 5B). The analysis revealed that conditions were significantly different from one another. Notably, culturing the cells in the presence of both SP-D (1 μg/ml) and GM-CSF (10 ng/ml) significantly reduced the formation of granulomas, compared to GM-CSF alone (p < 0.05; Figure 5B). Together, these results suggested that LPI BAL cells form granulomatous structures spontaneously, and GM-CSF enhances their formation. The data also suggest that SP-D can reduce GM-CSF mediated increase in granuloma formation (p < 0.05).

## Discussion

PAP in LPI is often untreatable and results in death. In this study, we carefully characterized BALF and tissue samples obtained from an LPI patient. The data revealed elevated levels of protein and dying cells in the airways. In addition, large cholesterol crystals and abnormal tubular myelin structures were found. Our primary cell culture assays using Alexa-647 conjugated BSA and apoptotic Jurkat T cells showed that pre-incubation of the LPI BAL cells with SP-D and GM-CSF increased their innate immune functions. Surprisingly, many of these LPI cells, but not the control cells, became elongated and stopped internalizing foreign material; eventually they formed granulomas *ex vivo*. Notably, treating these cells with GM-CSF *ex vivo *dramatically increased granuloma formation. However, treating the cells with SP-D reduced GM-CSF-mediated granuloma formation. Therefore, these findings may provide important clues for devising better treatment of PAP in LPI patients.

Although various SLC7A7 mutations can cause LPI, the importance of different mutations in the disease phenotype is not clearly established [45]. LPI is relatively frequent in Finland and Italy [46,47], and in Japan, 1:119 individuals are heterozygous carriers for this LPI gene mutations with estimated frequency of 1:50000 individuals living with LPI [48]. LPI has also been reported in other countries where variable penetrance of specific founder mutations was shown to be responsible for the disease [37,49]; hence, many LPI individuals with SLC7A7 mutations may be misdiagnosed.

This is the first report that shows LPI in a patient with Dutch ancestry. Consistent with the definition of PAP [1,12,27], this patient's BALF had PAS positive material (data not shown) and >100-fold protein levels compared to control, as well as increased numbers of dead cells (Figure 1). High amount of cholesterol and several cholesterol crystals were also seen (Figure. 2). These pathological indices are consistent with reduced lung function seen in these patients.

Although Western blot analyses showed that SP-D was present in the BALF, some SP-D was modified or tightly bound to other components. Furthermore, a considerable amount of SP-D was partially cleaved (Figure 1D). These conditions would render SP-D less functional [50,51]. Furthermore, electron microscopy analysis of the surfactant lipid pellets showed alterations in tubular myelin structures; notably, the tubular myelin had circular lattice structures, as opposed to the SP-A-mediated square lattice-like structures that are typically seen in healthy humans (Figure 1E and 1F) [43,52]. Tubular myelin with circular lattice structures is not reported in normal human lungs, but could be generated with SP-D and phosphatidylinositol, *in vitro *[43]. Since these lipid structures could trap SP-D, together with the fact that there was degraded SP-D, we suggest that the bioavailability of free SP-D is limited in the airways of PAP lungs.

Electron microscopy analysis further revealed the presence of large numbers of cholesterol crystals in the surfactant consistent with previous reports on LPI with PAP [32,33,39]. Since the cholesterol level is elevated in airways and serum of idiopathic PAP patients [53], cholesterol may be one of the important contributors of the pathology in all types of PAP. High cholesterol levels in the lung surfactant increases surface tension [54,55], and thus may reduce overall lung function. Interestingly, lovastatin, a cholesterol-reducing drug, has been shown to increase apoptotic cell clearance [56]. Therefore, we suggest that it would be worthwhile to explore the possibility of lowering cholesterol in the lungs while increasing dead cell clearance. This may be an alternative treatment option for reducing pulmonary complications in LPI patients.

Since GM-CSF [2,3,22,23,57-62] and SP-D deficiency [24-26,42] cause a PAP-like phenotype, we examined their potential therapeutic role *ex vivo*. Although both GM-CSF and SP-D are usually present in the lungs, it is important to determine whether the BAL cells isolated from the patient respond to these proteins. We were able to show a positive effect of GM-CSF and SP-D in the uptake of protein and apoptotic cells (Figure 3). These results are consistent with the roles of GM-CSF and SP-D suggested by *in vivo *mouse experiments [30,63,64]. Therefore, our results show that, similar to wild type cells, "healthy" LPI cells can respond to SP-D and GM-CSF effectively.

These healthy viable LPI cells however also formed granulomatous structure *ex vivo *(Figure. 5). Within about one week, many of these cells become elongated and large. They stopped internalizing foreign materials regardless of the presence or absence of SP-D or GM-CSF (Figure 5). Some of the cells formed elongated fibers and connected with each other whereas others migrated to a common location and formed granulomatous structures. It is logical to consider that defective surfactant homeostasis [1-7,22-26,42,57-62,65-68], accumulation of dead/dying cells [30] and foamy macrophages [21,25,26,69], and increased granulomas [39] would reduce lung function in the patients with PAP and LPI. Therefore, alternative strategies are necessary to treat PAP in these patients.

Aerosol [70,71] and subcutaneous [14,72] GM-CSF therapy have been successful in treating some patients with PAP [14,70-72]. Overexpression of GM-CSF in mouse [20,73] and rat [74,75] lungs has been shown to increase the recruitment of monocytes [20,75] and proliferation [73] and differentiation of alveolar macrophages [20]. Under certain conditions, GM-CSF also increases granuloma formation [75] and fibrosis [74] in the lung. Subcutaneous GM-CSF therapy (6 μg/kg per day) has been tried for treating PAP in another child with LPI, but was unsuccessful because the administration of GM-CSF had side effects and lead to excessive leukocytosis [34]. A recent report described a successful therapeutic WLL on a 10-year-old Italian boy with LPI who presented with PAP [46]. The pulmonary condition in the patient described in our report, however, did not improve after WLL, and the patient eventually died of pulmonary insufficiency.

## Conclusions

Our experiments using the primary cells isolated from the LPI patient did not confirm the effectiveness of GM-CSF alone in protein or apoptotic cell uptake assays; however, when GM-CSF was added together with SP-D, the innate immune potential of these LPI cells were enhanced. GM-CSF also showed a remarkable increase in granulomatous structure formation, but SP-D was able to reduce the negative effect of this cytokine. Taken together, our results suggest that although GM-CSF is a possible therapeutic molecule for treating patients with autoimmune idiopathic PAP, it may not be suitable for treating PAP in LPI patients. We suggest that alternative approaches (e.g., statins) need to be considered for clearing dying cells and cholesterol from the airways of these patients.

## Abbreviations used

PAP: pulmonary alveolar proteinosis; LPI: lysinuric protein intolerance; SP-D: surfactant protein D; GM-CSF: granulocyte/monocyte colony stimulating factor; BALF: bronchoalveolar lavage fluid; WLL: whole lung lavage.

## Competing interests

The authors declare that they have no competing interests.

## Authors' contributions

DND performed experiments and drafted the manuscript. NF performed experiments in this manuscript. SD performed BAL procedure and participated in manuscript preparation. HG participated in data interpretation and manuscript preparation. NP devised the study and participated in the interpretation of data and manuscript preparation. All authors read and approved the final manuscript.
